# Functional promoter -31G/C variant of *Survivin* gene predict prostate cancer susceptibility among Chinese: a case control study

**DOI:** 10.1186/1471-2407-13-356

**Published:** 2013-07-24

**Authors:** Jiawei Chen, Xinhai Cui, Hai Zhou, Chao Qin, Qiang Cao, Xiaobing Ju, Pu Li, Hongzhou Cai, Jian Zhu, Xiaoxin Meng, Meilin Wang, Zhengdong Zhang, Pengfei Shao, Jie Li, Changjun Yin

**Affiliations:** 1 State Key Laboratory of Reproductive Medicine, Department of Urology, The First Affiliated Hospital of Nanjing Medical University, Nanjing, 300 Guangzhou Road, Nanjing 210029, China; 2 Department of Pediatric surgery, Qiluhospital Shandong University, Jinan, China; 3 Department of Molecular and Genetic Toxicology, School of Public Health, Nanjing Medical University, Nanjing, China

**Keywords:** Prostate cancer, Genetic variation, *Survivin*, Apoptosis

## Abstract

**Background:**

Abnormal expression of Baculoviral inhibitor of apoptosis repeat-containing 5 (*BIRC5*, also called as *survivin*), a novel member of the inhibitor of apoptosis protein (IAP) family, has implications in many types of cancer and is considered as a new therapeutic target. We suppose that genetic variant rs9904341 in the 5′ UTR region of *survivin* gene may be associated with the development and progression of prostate cancer (PCa) in Chinese population.

**Methods:**

TaqMan assay method was used to genotype the polymorphism in the hospital-based case–control analysis of 665 patients with PCa and 710 age-matched cancer-free controls. The genetic associations with the occurrence and progression of PCa were calculated by logistic regression.

**Results:**

Our results indicated that compared with GG genotypes, there was a statistically significant increased risk of PCa associated with those with CC genotypes [odds ratios (ORs) = 1.57, 95%confidence intervals (CIs) = 1.17-2.13, *P* = 0.004]. Moreover, stratification analysis revealed that the association was more pronounced in subgroups of nondrinkers, nonsmokers and those without a family history of cancer (all *P* < 0.05). In addition, we observed that PSA ≥ 20 was more frequent in patients carrying GC/CC genotypes than in those with a wild type genotype.

**Conclusion:**

The functional *survivin* rs9904341 genetic variant may have a substantial influence on the PCa susceptibility and evolution.

## Background

Apoptosis is an essential genetic session which is necessary for the proper development of an organism [1]. The accumulation of virtually immortal cells,as the result of apoptosis evasion, leads to many human disorders or even cancer, which facilitates the acquisition of further molecular aberrations associated with biologically aggressive behaviors [2], As the apoptosis regulator containing four exons separated by three introns spanning 14.7-kb, *surivivin* (encoded by *BIRC2*) -is the smallest member of inhibitory apoptosis protein (IAP) family [3,4]. It has attracted great attention for its unique, bifunctional role in inhibiting apoptosis and cell cycle regulation [5,6]. *Survivin* also regulates the apoptosis pathway by suppressing the initiator caspase-9 and effector caspase-3 and caspase-7 [7-9]. Numerous studies have demonstrated that the reduction of *survivin* expression could bring with activated caspase, spontaneous apoptosis, and inhibition of cell proliferation and tumor growth, while the overexpression of *survivin* appeared to be uniquely associated with the inhibition of apoptosis [10]. Besides the anti-apoptotic properties, *survivin* expressed in a cell cycle-regulated manner regulates the cell division. It is abundantly expressed in the G2/M phase of the cell cycle, supporting the rapidly dividing cell mechanism, [6,11]. Furthermore, animal studies supported that *survivin* in conditional knockout mice had shown phenotypes with exaggerated apoptosis, with or without catastrophic mitotic defects [12-16].

Prostate cancer (PCa) is a frequently-occurring disease and is the second leading cause of cancer-related deaths of Western males. An analysis of the incidence and mortality of prostate cancer patients showed that approximately 217,730 new cases occurred and 32,050 deaths in 2010 in the U.S. [17]. The incidence rates of PCa in China and America vary substantially, with a much lower morbidity observed in China than in America. However, the occurrence of PCa is rapidly increasing in recent years in China [18].

Previously, accumulating evidences revealed that the expression of *survivin* was invariably up-regulated in human cancers, associated with the resistance to radiation or chemotherapy therapy, and poor prognosis [19-23].As to PCa, several studies identified that the *survivin*, though not normally expressed in the normal tissue and secretory epithelial cells of the prostate [24], was strongly expressed in PCa tissues and PCa cell lines at the level of RNA or protein [25-29]. Moreover, substantial evidence indicated that overexpression of *survivin* was related to the established features of biologically aggressive PCa, such as Gleason score and metastases [30-32]. As a whole, *survivin* appears to be the most promising diagnostic and prognostic markers in monitoring PCa.

Given the significant role of *survivin* in PCa, with biological, prognostic and therapeutic implications, we hypothesized the functional single nucleotide polymorphisms (SNPs) in the *survivin* gene in charge of expression or activity might contribute to the susceptibility and survival of PCa. Recently, the possible associations between the SNPs in the *survivin* gene and various types of cancer have been investigated in some prior studies [33-43]. Among them, we paid more attention to one SNP −31 G/C (rs9904341) in the promoter of *survivin* located at the cell cycle dependent elements (CDE) and cell cycle homology regions (CHR) repressor binding site [44]. Transcriptional expression of *survivin* gene might be modulated by CDE/CHR elements, as potentially implicated in imparting cell cycle periodicity of expression in G2/M [6]. To verify this hypothesis, we choose the genotype *survivin* rs9904341 polymorphism in our study of PCa in Chinese to identify if genetic variants in *survivin* gene may result in the susceptibility and progression of PCa.

## Methods

### Study population

Between September 2003 and January 2010, unrelated male (n = 1375) self-described ethnic Han Chinese were consecutively enrolled from the First Affiliated Hospital of Nanjing Medical University, Nanjing, China. All of the incident sporadic PCa cases (n = 665) were newly diagnosed, histopathologically confirmed and identified by reviewing the medical records that they had no prior history of other cancers. Healthy controls (n = 710) consisted of randomly-selected volunteers, and if they were unrelated cases and age-matched were also ascertained at the First Affiliated Hospital of Nanjing Medical University. Before enrollment, peripheral blood was obtained from every individual. A standard questionnaire through face-to-face interviews by trained interviewers was made to collect demographic data and related factors, including age, race, smoking history, alcohol intake, and family history of cancer. The study was approved by the Institutional Review Board of the Nanjing Medical University, Nanjing, China. At recruitment, written informed consent was obtained from all participants involved in this study. Those subjects who smoked less one cigarette per day and less one year over their lifetime were defined as nonsmokers and the rest as smokers. Lighter or heavier smokers were classified by WHO (World Health Organization. Guidelines for he Conduct of Tobacco Smoking Surveys for the General Population. Geneva, Switzerland: World Health Organization; 1983. Document WHO/SMO/83.4.). The participants who had pack-years value (cigarettes per day/20) × (years smoked) < 20 were considered as lighter smokers, the others (pack-years value ≥ 20 pack-years) were considered as heavier smokers. Those who drunk at least three times per week for more than 6 months were defined as drinkers; otherwise, they were considered as nondrinkers. Family history of cancer was defined as any occurrence of cancer in first-degree relatives (parents, siblings, or children). Disease stage was determined by clinical stage (all cases were classified according to the TNM classification system), Gleason score and PSA value. Localized prostate cancer could be detectable clinically on examination, but had not proliferated out of the prostate (T_1-2_N_0_M_0_). Advanced cancer meant the cancer had spread through the prostatic capsule (T_3-4_N_X_M_X_ or T_X_N_1_M_X_ or T_X_N_X_M_1_). The Gleason score was estimated by pathologists in the hospital by using the Gleason scoring system. Based on the EAU Guidelines on Prostate Cancer and D’Amico’s Risk-Based management of PCa, serum PSA value was classified into two groups PSA > 20 ng/ml and PSA ≤ 20 ng/ml. The participation response rates for both case and control subjects were > 85%.

### DNA extraction and polymorphism genotyping

Genomic DNA was extracted from the peripheral blood lymphocytes by using the conventional phenol-chloroform method. SNP rs9904341 G > C in the promoter of *survivin* gene was genotyped by the TaqMan MGB method with ABI 7900HT Real Time PCR system (Applied Biosystems, Foster city, CA) according to the manufacturer’s instructions. The primers and probes for rs9904341 G > C as follows: Forward primer, 5′- CGTGCGCTCCCGACAT-3′, reverse primer, 5′- GATGCGGTGGTCCTTGAGAA-3′; Probe G, 5′- FAM-TGAATCGCGGGACC-MGB-3′, Probe C, 5′- HEX-TTGAATCGCCGGACC-MGB-3′. Amplification was executed in 5 μL volumes in the 384-well plate, for 2 min at 50°C,10 min at 95°C, followed by 45 cycles of 95°C for 15 sec and 60°C for 1 min . The ABI 7900HT Real Time PCR system was adopted by the genotyping assay. The SDS 2.4 software was used to automatically collect and analyze the data and to subsequently generate the genotype calls in a blind manner. Four negative controls in each 384-well plate were used for quality control. Samples making up more than 5% were randomly chosen for repeated genotyping, yielding a 100% concordant.

### Statistical analysis

Deviation of genotype distribution from the Hardy-Weinberg equilibrium (HWE) for SNP rs9904341 amongst controls was calculated by a goodness-of-fit χ^2^ test. The student’s t-test (for continuous variables) or chi-square test (for categorical variables) was performed to estimate the differences in frequency distributions of selected demographic variables, selected variables, and frequencies of genotypes between cases and controls. The associations between *survivin* SNP rs9904341 and PCa risk were assessed by computing odds ratios (ORs) and 95% confidence intervals (CIs) from unconditional logistic regression analysis with or without the adjustment for potential confounders. The P value < 0.05 was the criterion of statistical significance, and all of the statistical tests were two sided. All of the statistical analyses were dealt with SAS 9.1.3 software (SAS Institute, Cary, NC, USA), unless indicated otherwise.

## Results

### Characteristics and clinical features of study population

The demographic characteristics and the clinical information of 665 PCa patients and 710 controls in the study were outlined in Table 1. Briefly, there was no significant difference in terms of distribution of age between cases and controls (*P* = 0.833). However, in comparison with the controls, a crucially higher proportion of the PCa patients smoked (57.9% versus 50.3%, *P* = 0.005), drunk (29.6% versus 24.4%, *P* = 0.028) and had family history of cancer (19.4% versus 7.8%, *P* < 0.001). Specially, the risk of cigarette addictives (> 20 pack-years) was 1.38 times more (95%CI, 1.20-1.61) than nonsmokers. For 40.9% of these 665 patients, PSA ≤ 20 ng/ml, and for the rest, PSA were > 20 ng/ml. 390 of them were defined as localized stage cancer, but only 275 of 710 had the advanced stage cancer. When stratified according to Gleason score, the percent of Gleason score < 7, = 7 and > 7 was 33.8%, 33.2% and 33.0%, respectively. These variables were further adjusted with multivariate logistic regression models.

**Table 1:** Demographic and clinical variables of prostate cancer cases and controls

**Variables**	**Cases (n = 665)**		**Controls (n = 710)**		P ^*^
	**n**	**%**	**n**	**%**	
Age (years) (Mean ± SD)	71.4 ± 8.0		71.3 ± 7.4		0.833
≤ 71	310	46.6	355	50.0	0.210
> 71	355	53.4	355	50.0	
Smoking status					0.005
Never	280	42.1	353	49.7	
Ever	385	57.9	357	50.3	
Pack-years of smoking					<0.001
0	281	42.3	353	49.7	
0-20	127	19.1	177	24.9	
>20	257	38.6	180	25.4	
Drinking status					0.028
Never	468	70.4	537	75.6	
Ever	197	29.6	173	24.4	
Family history of cancer					<0.001
No	536	80.6	655	92.2	
Yes	129	19.4	55	7.8	
Clinical stage					
Localized	390	58.7			
Advanced	275	41.3			
Gleason score					
<7	225	33.8			
=7	221	33.2			
>7	219	33.0			
PSA (ng/ml)					
≤20	272	40.9			
>20	393	59.1			

^*^ T-test for age distributions between the cases and controls; two-sided χ^2^ test for others selected variables between the cases and controls.

### Genotype and allele frequencies of *survivin* polymorphism in cases and controls

Genotyping call rate of rs9904341 was 99.25%. The genotype and allele distributions of *survivin* SNP rs9904341 between PCa cases and controls were summarized in Table 2. The observed genotype frequencies of SNP rs9904341 among the controls confirmed to the HWE (*P* > 0.05). We observed that frequencies of *survivin* genotypes were significantly different between cases and controls (*P* = 0.014 and 0.003 for genotype and allele, respectively), which principally derived from discrepancy between wild-type GG (22.5% for cases and 28.9% for controls) and variant-type CC (29.5% for cases and 24.5 for controls). In the multivariate logistic regression models with adjustments of age, smoking status, drinking status, and family history of cancer, our results indicated that CC genotype was associated with a significantly increased risk of PCa compared with the GG genotype (OR = 1.57, 95% CI = 1.17-2.13).

**Table 2:** **Genotype and allele frequencies of the *BIRC5 *rs9904341 polymorphisms among the PCa cases and controls**

**Polymorphisms**	**Cases (n = 665)**		**Controls (n = 710)**		P *	**Adjusted OR (95%CI) **^**†**^
	**n**	**%**	**n**	**%**		
*BIRC5* (rs9904341)						
GG	150	22.5	205	28.9	0.014	1.00 (reference)
CG	319	48.0	331	46.6	0.038	1.32 (1.01-1.72)
CC	196	29.5	174	24.5	0.004	1.57 (1.17-2.13)
CG + CC	515	77.5	505	71.1	0.008	1.40 (1.09-1.80)
G	619	46.5	741	52.2	0.003	1.0 (reference)
C	711	53.5	679	47.8		1.12 (1.04-1.20)
*P*_*trend*_					0.004	

^*^ Two-sided χ^2^ test for the either genotype distributions or allele frequencies between the cases and controls.

^†^ Adjusted for age, smoking status, drinking status, and family history of cancer in logistic regression model; 95% CI: 95% confidence interval; OR: odds ratio.

### Stratification analyses

Stratification analyses of age, smoking status, drinking status, pack-years of smoking and family history of cancer with the genotypes of *survivin* rs9904341 polymorphism were presented in Table 3. We found that individuals with CC/GC genotypes had a significantly increased risk of PCa than those with GG genotype, which appeared to be more evident in nonsmokers and nondrinkers, and negative in those with family history of cancer. To further evaluate the influence of the genotype on the severity of PCa, we studied the association between rs9904341 polymorphism and clinicopathological characteristics of PCa patients. As shown in Table 4, the rs9904341 GC/CC genotypes were much more in PCa patients with PSA value > 20 ng/ml (*P* = 0.01, adjusted OR = 1.62, 95% CI = 1.12-2.33). However, no association was observed between the evaluated genotypes and subgroups with clinical stage and Gleason score (all *P* > 0.05).

**Table 3:** Stratification analyses between the genotypes of rs9904341 G > C polymorphism and risk of PCa

**Variables**	**Cases/Controls**	**Genotypes(Cases/Controls)**				P*	**Adjusted OR(95%CI) **^**†**^
		**GG**		**GC + CC**			
		**n**	**%**	**n**	**%**		**GC + CC versus GG**
Total	665/710	150/205	22.5/28.9	515/505	77.5/71.1	**0.008**	**1.40 (1.09-1.80)**
Age							
≤ 71	310/355	66/98	21.3/27.6	244/257	78.7/72.4	0.060	1.35 (0.93-1.95)
> 71	355/355	84/107	23.7/30.1	271/248	76.3/69.9	0.052	1.44 (1.02- 2.01)
Smoking status							
Never	280/353	62/105	22.1/29.8	218/248	77.9/70.2	**0.031**	**1.47 (1.01-2.12)**
Ever	385/357	88/100	22.9/28.0	297/257	77.1/72.0	0.109	1.35 (0.96-1.89)
Pack-years of smoking							
0-20	128/177	37/49	28.9/27.7	91/128	71.1/72.3	0.899	0.94 (0.52-1.57)
> 20	257/180	52/51	20.2/28.3	205/129	79.8/71.7	0.050	1.56 (0.99-2.58)
Drinking status							
Never	468/537	109/159	23.3/29.6	359/378	76.7/70.4	**0.024**	**1.37 (1.02-1.83)**
Ever	197/173	41/46	20.8/26.6	156/127	79.2/73.4	0.191	1.46 (0.89-2.38)
Family history of cancer							
No	536/655	121/187	22.6/28.6	415/468	77.4/71.4	**0.019**	**1.36 (1.04-1.77)**
Yes	126/55	29/18	22.5/32.7	100/37	77.5/62.3	0.145	1.60 (0.79-3.25)

*Two-sided χ^2^ test for either genotype distributions or allele frequencies between the cases and controls.

^†^ Adjusted for age, smoking status, drinking status and family history of cancer in logistic regression model; 95% CI: 95% confidence interval.

**Table 4:** **Associations of the the *BIRC5 *rs9904341 polymorphisms with the clinicopathological characteristics of PCa**

**Variables**	**Genotypes, n (%)**		P ^*^	Adjusted OR (95%CI) ^*^
	**GG**	**CG + CC**		**CG + CC versus GG**
Clinical Stage^†^			0.260	
Localized	94 (24.1)	296 (75.9)		1.00 (reference)
Advanced	56 (20.3)	219 (79.6)		1.23 (0.85-1.79)
Gleason Score			0.534	
<7	55 (24.4)	170 (75.6)		1.00 (reference)
=7	51 (23.1)	170 (76.9)		1.10 (0.71-1.71)
>7	44 (20.1)	175 (79.9)		1.26 (0.80-1.98)
PSA(ng /ml)			0.010	
≤20	75 (27.6)	197 (72.4)		1.00 (reference)
>20	75 (19.1)	318 (80.9)		1.62 (1.12-2.33)

^*^ The *P* value and ORs were calculated and adjusted for age, smoking, drinking, and family history of cancer in logistic regression model; 95% CI: 95% confidence interval; OR: odds ratio.

^†^Localized: T_1- 2_N_0_M_0_; Advanced: T_3-4_N_x_M_x_ or T_x_N_1_M_x_ or T_x_N_x_M_1_. Clinical staging according to the international TNM system for PCa.

## Discussion

Currently, the role of SNP rs9904341 in the promoter region of *survivin* is studied to make clear of the susceptibility and manifestation of clinicopathological characteristics of PCa in Chinese population. This is the first investigation done so far to evaluate the role of the SNP (rs9904341) in the *survivin* gene pertaining to the risk and progression of PCa.

Our results revealing the association between this *survivin* polymorphism and the risk of PCa are biologically plausible. It is broadly accepted that PCa’s resistance to apoptosis is associated with an alteration in the expression of pro-apoptotic [45] and anti-apoptotic protein [46]. *Survivin*, as an apoptotic inhibitor, plays a significant role in the apoptosis pathway and cell proliferation [6]. Growing evidence enhanced that *survivin* was prominently over-expressed in various human cancers [20], paralleling with the deregulated apoptosis in cancer [7]. With respect to PCa, Krajewska *et al.* observed that increased IAP (including *survivin*) expression occured early in the pathogenesis of PCa [28]. Koike *et al.* demonstrated that *survivin* was associated with PCa cell proliferation [29]. In addition, some studies suggested that *survivin* made a crucial contribution to apoptotic resistance in PCa either in vitro or in vivo [26,47].

Numerous epidemiological studies, such as case–control, cohort and genome wide association studies (GWAS), have exhibited the role of low-risk genetic variants in susceptibility to PCa. In our present research, an increased risk of PCa was observed among the individuals carrying C allele compared with those carrying G allele, as evidenced by the data obtained in previous studies of urothelial carcinoma [39], nasopharyngeal carcinoma [33], esophageal cancer [37], colorectal cancer [40], endometrial cancer [48] and gastric cancer [41]. What mechanism underlies the association between this SNP and susceptibility of PCa is still not well-known. It might be safe to suppose that rs9904341 SNP, located at the binding site for the CDE/CHR repressor in the promoter of *survivin*, affects transcriptional activity by modifying the binding motif of the CDE/CHR repressor which leads to the measurable and functional discrepancy in *survivin* expression. This supposition has been vigorously advocated by other investigators. Xu et al. firstly demonstrated that the presence of mutation including rs9904341 polymorphism was correlated with increased *survivin* expression at both mRNA and protein levels in some cell lines [44]. Jang et al. observed that -31C allele had a significantly higher transcriptional activity compared with -31G allele in vitro promoter assay for lung cancer [42]. Nikiteas et al. revealed that mRNA levels of *survivin* expressed by homozygous for the -31CC *survivin* genotype were approximately 1.6 times higher than those with the GG/GC genotypes [40]. Habuchi et al. reported that a significantly higher *survivin* mRNA and protein expression level was observed in bladder cancer cell with an increased number of -31C allele by immunohistological evaluation and reverse transcriptase-PCR [36]. Taking these observations into consideration, we assumed that *survivin*g probably contributed to the susceptibility of PCa, and our finding confirmed this hypothesis. Moreover, according to the Hapmap database, there was another polymorphism rs8073069 in complete linkage disequilibrium with rs9904341, so we only selected rs9904341 to genotype and analyze.

It is well known that PCa is a complex malignancy caused by multifactor: age, gene or environment. In stratified analyses, we observed that the effect of rs9904341 polymorphism on the risk of PCa was overstressed among nondrinkers, nonsmokers and those without a family history of cancer, indicating that this polymorphism was an independent risk of PCa and the interaction of gene-environment might be very weak in the subgroups. Furthermore, a line of studies have manifested that the over-expression of *survivin* was considered to be a marker for aggressive PCa and signaling a poor prognosis [30-32]. According to the subgroup analyses by clinicopathological characteristics, our results detected that PCa patients having the genotypes GC/CC were significantly associated with cases whose PSA value > 20 ng/ml, suggesting that the polymorphism appeared to play an important role in the progression of PCa. Our conclusion is compatible with the results of one previous research by Wang et al., but contradictory to the conclusions of other studies [36,49-51]. The discrepancy of these findings may be elucidated by the diverse molecular mechanisms of carcinogenesis in tumors, rather merely by the different genetic background.

Interestingly, our results showed that the genotype distributions of the *survivin* polymorphism vary with ethnicity compared with the published data. In the controls of our study, the frequencies of GG, GC, CC genotypes in rs9904341 are 28.9%, 46.6%, 24.5%, respectively, which is similar to the date derived from some previous studies on Asian population [36,38,41,42,49], but different from the results reported by Bayram and Veress in European [34,43]. Probably, this difference results from the genetic discrepancy. Therefore, our findings should be independently validated in other communities with high incidence, especially in Caucasian.

Additionally, we are aware of a few limitations in our study, some of which cannot be overcame . First, -based on the patients and the control group randomly from the same hospital, we cannot completely rule out the inherent selection bias. Nevertheless, in order to minimize potential biases, we have made a rigorous epidemiological design of study subjects and more statistical adjustments for known risk factors. Second, our study lacks detailed information including environmental exposure and survival data, and our sample is medium-sized, which may weaken the statistical power of this study. Under the current sample size, we have 80% power at a 0.05 significance level to detect an OR of 1.4 or higher and 0.69 or lower with an exposure frequency of 24.5%. In addition, our conclusions are in accordance with the meta analyses (3329 cases and 3979 controls) in regard to the association between rs9904341 polymorphism and the risk of cancer performed by Mittal [52]. Third, our findings together with other observations from literature are still in conflict, and the significant association between rs9904341 polymorphism and PCa risk should be interpreted with caution.

## Conclusions

In summary, our current results provide the primary evidence that the polymorphism rs9904341 in the promoter of *survivin* is a genetic susceptibility factor for the pathogenesis and progression of PCa in Chinese population. The observations of the present study are valuable to improve our understanding of the role of *survivin* in PCa. However, it is necessary that additional studies with more detailed data on environmental exposure and survival data of more samples and functional characterizations are needed to confirm our results, particularly in PCa.

## Competing interests

The authors declare that they have no competing interests.

## Authors’ contributions

JC, CQ, QC, PS, JL, CY were responsible for the study design. JC, XC, HZ, PL, JZ, HC were involved in data acquisition and analysis and performed the Taqman experiments. Statistical analyses, data interpretation and manuscript drafting were done by CQ, XJ, XM, MW, ZZ. All authors critically reviewed and approved the final manuscript.

## Pre-publication history

The pre-publication history for this paper can be accessed here:

http://www.biomedcentral.com/1471-2407/13/356/prepub
